# Identification of an unrecognized circRNA associated with development of renal fibrosis

**DOI:** 10.3389/fgene.2022.964840

**Published:** 2023-01-04

**Authors:** Yun Zhu, Weimin Yan, Shuangyan Xu, Xiaochao Yu, Shuo Sun, Shaoxiong Zhang, Ran Zhao, Jiayue Tao, Yunwei Li, Cuie Li

**Affiliations:** ^1^ Department of Dermatology, The People’s Hospital of Yuxi City, Yuxi, China; ^2^ Graduate School, Kunming Medical University, Kunming, China; ^3^ Department of Urology, The Third Hospital of Shandong Province, Jinan, China; ^4^ Department of Geriatrics, The People’s Hospital of Yuxi City, Yuxi, China

**Keywords:** renal fibrosis, circRNA_0002158, fibronectin, PAI-1, HK-2

## Abstract

**Backgroud:** Renal fibrosis is the common characteristic of chronic kidney disease. Circular RNA plays an essential role in the occurrence and development of Renal fibrosis, but its regulative mechanism remains elusive.

**Methods:** The animal and cell model of Renal fibrosis was established, and RNA-sequencing and real-time polymerase chain reaction (qRT-PCR) experiments were implemented. Subsequently, experiments for detecting apoptosis and proliferation of cell, were carried out, and the isobaric tags for relative and absolute quantification proteomics analyses were performed accordingly.

**Results:** It was found that a newly discovered Circular RNA (circRNA_0002158), is highly expressed in kidneys or cells with fibrosis, implying that this Circular RNA might be associated with the occurrence and development of Renal fibrosis. Subsequently, the overexpression and knockdown of circRNA_0002158 were conducted in the human kidney epithelial cell line (HK-2) cells, and the results indicated that the circRNA_0002158 could inhibit apoptosis, and promote proliferation of cells. The kidney injury-related factors, including Fibronectin and plasminogen activator inhibitor-1 (PAI-1), were decreased in HK-2 cells with overexpression of circRNA_0002158, while the results were reversed in cells with knockdown of circRNA_0002158. Finally, to explore the regulative mechanism of circRNA_0002158, the iTRAQ proteomics analyses were implemented for the cell samples with OE of circRNA_0002158 and its control, it showed that multiple genes and functional pathways were associated with the occurrence and development of Renal fibrosis.

**Conclusion:** CircRNA_0002158 is associated with regulating Renal fibrosis, and may contribute to ameliorating the progression of Renal fibrosis in the future.

## Introduction

Renal fibrosis (RF), a common pathological manifestation of all types of chronic kidney disease (CKD) progressing to the end-stage (Okada et al., 1996; Zeisberg and Neilson, 2010), has become a worldwide public health problem imposing a heavy burden on the healthcare system (Coresh et al., 2007). The renal prognostic significance is associated with the extent of tubulointerstitial fibrosis (Grande et al., 2010). During the progression of RF, the renal tubular cells are transformed into myofibroblasts, and then excessive extracellular matrix, including collagens, Fibronectins (FN), laminin and glycosaminoglycan, is synthesized (Tang et al., 2018). Plasminogen activator inhibitor-1 (PAI-1) was expressed at low levels in healthy kidneys (Eddy and Fogo, 2006), but increased in RF disease (Lee et al., 2001; Paueksakon et al., 2002). The molecular mechanism underlying of RF remains unknown, and it is necessary to elucidate the molecular mechanisms of tubulointerstitial fibrosis and approaches to mitigate or reverse it.

Circular RNAs (circRNAs) are covalently closed RNA molecules generated by a backshearing process. CircRNAs have been implicated in a variety of diseases, including diabetes, neurological diseases, cardiovascular diseases, chronic inflammatory diseases and cancer (Yi et al., 2021). In the search for potential cures for these diseases, circRNAs are in the spotlight. CircRNAs have a variety of regulative functions, and some of them could be translated into proteins involved in gene regulation. For example, circ-E-CAD RNA activates EGFR signaling by encoding E-cadherin variants to induce the occurrence of glioma (Yi et al., 2021). Some circRNAs are involved in transcriptional regulation. For example, exon-intron circRNAs (EIciRNAs) related to RNA polymerase II, which are mainly localized in the nucleus, could interact with U-SnRNP for promoting gene transcription (Yi et al., 2021). CircRNAs were also associated with selective cleavage of premRNAs. The circURI1 could interact with heterogeneous nuclear ribonucleoprotein M (hnRNPM) to modulate alternative splicing of multiple genes involved in the process of cell migration, thus suppressing metastasis of gastric cancer (Yi et al., 2021).

The circRNAs can also act as a miRNA sponge to regulate gene expression, and be associated with occurrence and development of RF. It indicated that overexpression (OE) of circRNA_37492 attenuated the TGF-β1/UUO-induced RF *via* targeting the miR-7682-3p/Fgb axis, while the homologous hsa_circRNA_0012138, as a possible competing endogenous RNA (ceRNA), was speculated to regulate multiple gene expressions in RF, suggesting that the circRNA_37492/hsa_circ_0012138 might act as a potential therapeutic target for obstructive RF (Cheng et al., 2022). The circRNA_30032 was mediated UUO-induced and TGF-β1-induced RF by sponging miR-96-5p to up-regulate the expression of profibrotic proteins, including FN, indicating that this cirRNA could induce RF *via* the miR-96-5p/HBEGF/KRAS axis (Yi et al., 2021).

In this study, to explore the molecular mechanism of RF, the RF model of rat was established successfully, and RNA-sequencing (RNA-seq) and real-time polymerase chain reaction (qRT-PCR) experiments were implemented. We identified a novel circRNA molecule (circRNA_0002158), which is expressed at lower levels in renal tissue of rat with RF than normal control. Therefore, it was speculated that circRNA_0002158 might play a critical role in the occurrence and progression of RF. Subsequently, we identified the homolog of circRNA_0002158 in the human, and generate a cell model of RF by using human kidney epithelial cell line (HK-2) cells according to the previous study (Tang et al., 2019). Furthermore, the experiments for detecting proliferation and apoptosis and proteomics analyses were implemented for the cell samples with OE of circRNA_0002158. Here we report the results.

## Materials and methods

### Cell culture and cell model of RF

The cell line (HK-2) was provided by Procell (Wuhan, China) and cultured in high glucose Dulbecco’s modified Eagle’s medium (Invitrogen, Carlsbad, United States). All media were supplemented with 10% fetal bovine serum (Gibco, Waltham, MA, United States) in the presence of 1% penicillin and streptomycin (Beyotime, Shanghai, China) under humidified atmosphere of 5% CO_2_ and 95% air at 37°C. HK-2 cells were treated with TGF-β1 (10 ng/ml) at 37°C for 72 h to generate a cell model of RF according to the previous study (Lopez-Hernandez and Lopez-Novoa, 2012).

### Animals and rat model of RF

The male Sprague Dawley (SD) rats (6–8 weeks) were purchased from the animal center of KunMing Medicine University (YunNan, China). All animal care and experiments were approved by the Institutional Animal Ethic Committee of KunMing Medicine University, and conducted in compliance with the requirements of the national act on the use of experimental animals (China).

20 SD rats (6–8 weeks) were randomly divided into RF and control (Ctrl) groups, with 10 ones in each group. The rats from RF group received unilateral ureteral obstruction (UUO) treatment to generate progressive RF (Chevalier et al., 2009), while the rats from Ctrl group were treated with sham operation. Rats were anesthetized with 2% pentobarbital (Shanghai Biochemical Co., Ltd, China), then the left ureter was exposed by a tilted incision, and it was obstructed by two-point ligations with 4–0 silk sutures. After abdominal incision was sealed with the same silk suture, the rats were returned to the cages. Sham-operated rats underwent the same procedure except ligation.

The rats from RF and Ctrl groups were sacrificed on the 20th day after the operation by routine single-cage feeding observation, free access to water and food, and the kidney tissue was collected. To assess model of RF, one portion of the kidneys from rat was fixed in 10% phosphate-buffered formalin, followed by paraffin embedding for histological staining. Hematoxylin and eosin (HE) staining was used to detect the degree of renal tissue injury, while Masson staining was implemented to detect the degree of fibrosis.

### RNA sequencing and analysis

Total RNAs were extracted from renal tissue samples using TRIzol reagent (Takara, Dalian, China), and the RNA samples were quantified after removal of DNA contamination. Subsequently, the integrity of the isolated RNAs were assessed with the Agilent 2100 Bioanalyzer (Applied Biosystems, Carlsbad, CA, United States). The ribosomal RNAs were removed from total RNAs. Then, strand-specific RNA-seq library preparation was carried out using the VAHTS Total RNA-seq (H/M/R) Library Prep Kit for Illumina^®^ (Vazyme, Nanjing, China) as previously (Zhao et al., 2019), and prepared cDNA libraries were sequenced in the HiSeq 4,000 system (Illumina, San Diego, CA, United States).

Undetermined and low-quality bases were removed and sequence quality was verified by FastQC2, and the reference genome was read map using Bowtie2 and Tophat2. CIRCExplorer and TopHat-fusion were utilized for *de novo* assembly of the mapped reads to circRNA and recognized back splicing reads in unmapped reads. All samples generated unique circRNA. Basd on reference data from the circBase database, circRNAs were identified in RNA-seq data by CIRCexplorer in current study. Finally, the circRNAs with a *p*-value < 0.05 and log_2_ fold change (FC) > 1 were considered differentially expressed (DE) circRNAs.

### Cell transfection

The plasmids containing circRNA_0002158 and control plasmids were obtained from GeneCreate (Wuhan, China). The siRNA circRNA_0002158 (sense: 5′-GCA AGG TTG ACA GAA CCT TC -3′) and siRNA circRNA_0002158–2 (sense: 5′-GTT GGC AAG GTT GAC AGA A-3′) were also synthesized by GeneCreate. The HK-2 cells were transfected with siRNAs or plasmids for 24 h using the Lipofectamine RNAi MAX transfection kit (Invitrogen, United States).

### Quantitative reverse transcription polymerase chain reaction (qRT-PCR)

The total RNAs were reverse-transcribed into complementary cDNA by RNA PCR Kit (TaKaRa, Dalian, China), and qRT-PCR was performed using the SYBR Premix Ex Taq Kit (Takara) and the 7,500 Real-time PCR System (Applied Biosystems, Foster, CA, United States) according to the manufacturer’s instructions. Glyceraldehyde 3-phosphate dehydrogenase (GAPDH) was used as the endogenous control for circRNA. Relative quantification was carried out using the comparative 2^−ΔΔCT^ method. Primers for circRNA_0002158, FN, PAI-1 and GAPDH were designed (Table 1) and synthesized by GeneCreate (Wuhan, China).

**TABLE 1:** The genes and primers used for qRT-PCR experiments.

Gene	Forward primer (5′-3′)	Reverse primer (5′-3′)
Fibronectin	TCA​CCA​CCA​CCA​GCA​CCA​G	AGA​CCC​AGG​AGA​CCA​CAA​AGC
PAI-1	GGC​TGG​TGC​TGG​TGA​ATG​C	CGGTGGGTGCTGGAGTCG
circ_0002158	GGT​AAT​GAG​ACG​GTG​CCT​GAG	TCT​GTC​AAC​CTT​GCC​AAC​TAT​CG
circ_0004883	CTC​AAC​TTG​TCT​CCA​ACT​TCA​TCA​C	TCT​GGG​AAA​CCT​TCT​GGA​AAT​GC
circ_0001606	CTG​GAC​CTT​TAT​CTG​GCT​TAC​TTT​G	TCC​GCT​TCT​CGT​GCT​TCT​TG
circ_0000756	CTT​GTG​TGG​ACA​TTG​ATG​AGT​GC	GTT​CGC​CTC​GCT​TCT​GAT​ACC
circ_0004474	CTA​CGG​CTA​CCT​GAA​GGA​CTA​TG	TAC​TGG​CTT​CTG​GCT​AGA​GGT​G
circ_0001106	TGC​CAG​GAG​TTC​ATT​ACA​AAT​CTT​C	ACA​TTG​CTG​TTA​GTG​CCA​TTG​C
circ_0000333	CAA​CAT​CAG​CAC​AGT​TCT​ATA​ATA​TCA​AG	CCT​CCC​TCA​GCA​AAG​TCA​TCC
GAPDH	AGG​TCG​GTG​TGA​ACG​GAT​TTG	GGG​GTC​GTT​GAT​GGC​AAC​A
Actin	TGG​ACT​TCG​AGC​AAG​AGA​TG	GAA​GGA​AGG​CTG​GAA​GAG​TG

### Western blotting (WB) analysis

Cultured HK-2 cells were harvested and homogenized using cell lysis buffer. Subsequently, the homogenates were centrifuged for 25 min at 4°C, 13,000 rpm, and the supernatants were collected accordingly. Protein amounts were evaluated with the BCA Protein Assay Kit (Beyotime, Shanghai, China). Equal amounts of protein samples were separated by denaturing 10% sodium dodecyl sulfate polyacrylamide gel electrophoresis (SDS-PAGE) and transferred onto polyvinylidene difluoride (PVDF) membranes.

The anti-Fibronectin antibody (Abcam, Cambridge,MA, United States), anti-GAPDH antibody (Abcam) and anti-PAI-1 antibody (Proteintech, Wuhan, China) were used. Membranes were incubated in a 5% skim milk TBST blocking solution at room temperature (RT) for 1 h and membranes were incubated with agitation at 4°C overnight with specific primary antibodies. Subsequently, membranes were incubated by secondary antibodies conjugated with horseradish peroxidase (HRP) (Proteintech) at RT for 45 min. At the last, protein bands were visualized using an enhanced chemiluminescence (ECL) Western blotting detection system (GE Healthcare, Amersham, United Kingdom).

### Cell viability detection

The viability of HK-2 cells was evaluated using the CCK-8 assay kit (Solarbio, Peking, China). The HK-2 cells were slightly seeded into the 96-well plates with 100 μL suspension per well overnight. At 0, 24, 48 and 72 h, 20 μL CCK-8 solution was added into each well, and then the plates were incubated for 2 h at 37°C. At last, absorbance was measured at 490 nm by microplate reader (Bio-Rad, Hercules, United States).

### Cell apoptosis detection

The apoptosis of HK-2 cells was analyzed using the luorescein Isothiocyanate (FITC) Annexin V Apoptosis Kit (BD Biosciences, San Jose, CA, United States) and flow cytometry according to the manufacturer’s guidelines. Briefly, cells cultured in 6-well plates were transfected at a concentration of 1 × 10^7^/ml for 72 h, were washed twice with PBS and resuspended cells in 1× Binding Buffer (10 mM HEPES/NaOH, pH 7.4, 140 mM NaCl, 2.5 mM CaCl_2_). Subsequently, 5 µL FITC Annexin V (BD Biosciences, San Jose, CA, United States) was put into the cell suspension, and they were incubated in the dark at RT, for 15 min. Finally, the cell suspension was mixed with 400 µL Annexin V Binding Buffer.

### Liquid chromatography-mass spectrometry (LC–MS/MS) and analysis

Briefly, the plasmids containing circRNA_0002158 was transfected into HK-2 cells for 48 h. Total protein was extracted and 100 μg proteins were digested with trypsin each sample.

MS assays were carried out according to the manufacturer’s protocol with minor modifications. The digested peptide mixture was dried and treated with 0.1% trifluoroacetic acid (TFA). Samples were iTRAQ labeled as follows: NC-1, X1; NC-2, X2; NC-3, X3; OE-1, X4; OE2, X5; and OE-3, X6. NC-1, NC-2 and NC-3 refer to three parallel groups of the empty vector-controlled normal control (NC) HK-2 cells, the other OE-1, OE-2 and OE-3 refers to three parallel groups of the same circRNA_0002158 overexpressed HK-2 cells, and X1, X2, X3, X4, X5 and X6 refer to the order from sample one to 6. And all of the labeled samples were mixed with equal amounts. After diluting the 5 μL samples, we used the Eksigent nanoLC system (SCIEX, United States) coupled to the TripleTOF 5600 + mass spectrometers to recover proteins and perform proteomic analysis of total proteins, which were identified using Proteome Discoverer 1.4 software (Thermo Fisher Scientific, MA, United States), and the resulting original file was imported into the UniProt KB/Swiss-Prot database for searching. The mass tolerances of the precursor and fragmentation were set to 10 ppm and 0.8 Da, respectively for the database search. Peptides with a false discovery rate  < 1% (q value < 0.01) and proteins with an area value lower than 1 × 10^6^ all were discarded. For the determination of differentially expressed proteins (DEPs), fold changes (FC) were calculated as the average comparison pairs among biological replicates. Proteins with FC larger than 1.2 and a *p* < 0.05 were considered to be changes with significant difference.

The DEPs were screened by LC–MS/MS were imported to Ingenuity Pathway Analysis (IPA) software. Then the Gene Ontology (GO, http://www.geneontology.org/) and Kyoto Encyclopedia of Genes and Genomes (KEGG) mapping of DEPs were analyzed to obtain enrichment pathways. Fisher’s exact test was used to calculate a *p*-value to determine the probability of each biological function.

### Statistical analyses

All data were presented as the mean ± standard deviation (SD) of three experimental repeats. Comparisons between two groups were analyzed using the two-tailed Student t-tests. For comparison among multiple groups, we used one-way ANOVA followed by Dunnett’s or Tukey’s *post hoc* test. A *p* < 0.05 was regarded to indicate a statistically significant difference. Statistical analyses were performed by SPSS Software (version 15.0) (IBM Corp.), and graphs were plotted by GraphPad Prism (version 9.12, San Diego, CA, United States).

### Availability of data

The datasets presented in this study had be submitted into the online repositories. The transcriptomic data could be obtained from NCBI with accession No: PRJNA895745; while proteomic data could be got from ProtemXchange with accession No: PXD037827.

## Results

### The circRNA_0002158 in animal and cell model of RF

**FIGURE 1:**
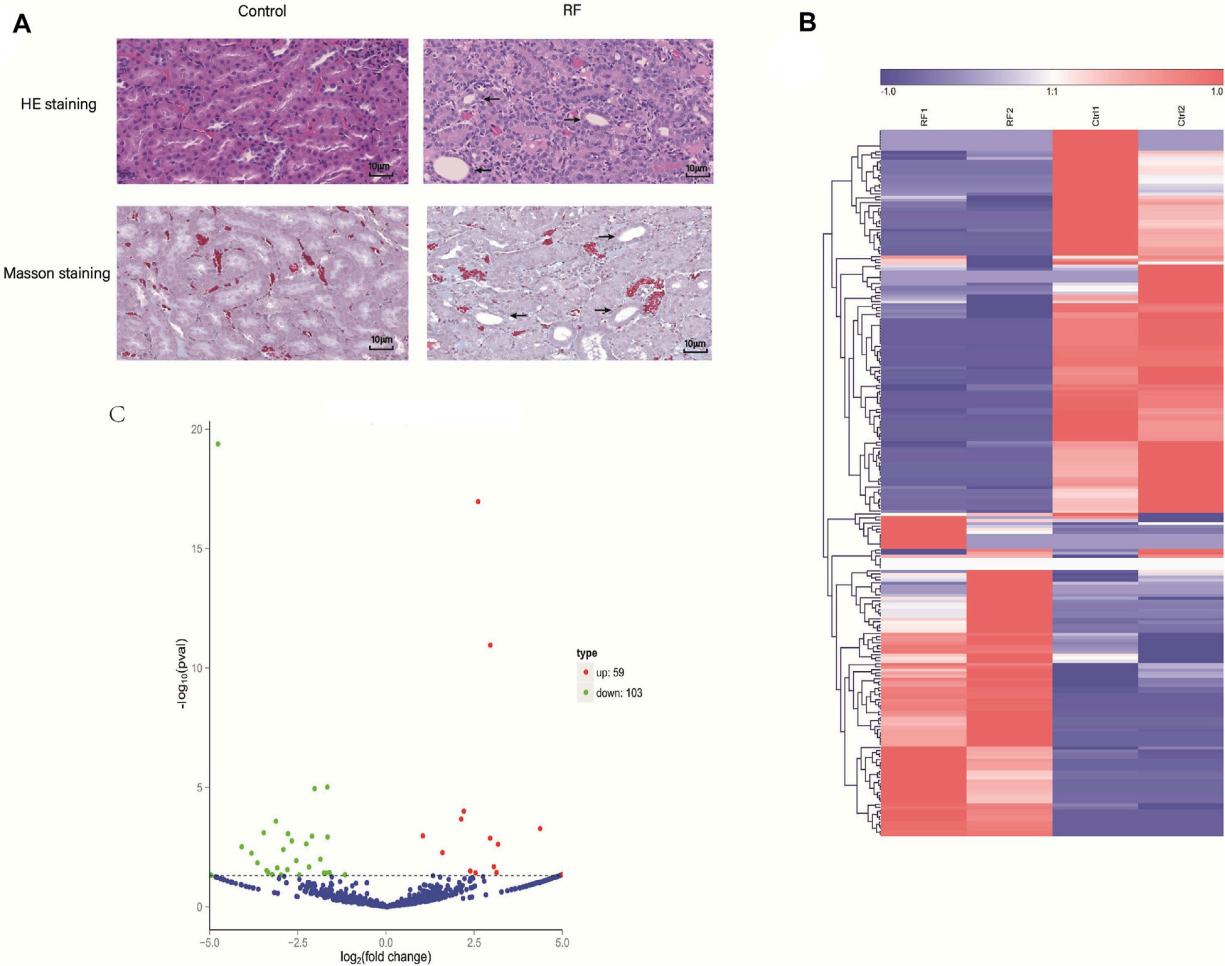
The establishment of renal fibrosis (RF) model of rats and exploration of cirRNAs expression. **(A)** Hematoxylin and eosin (HE) and Masson staining was used to detect the degree of injury and fibrosis of renal tissue, respectively. **(B)** The heat map indicates the differential expression of circRNAs between RF and Ctrl groups (2 samples for each group). The high and low expression of circRNAs are marked in red and green, respectively. **(C)** Volcano plots of circRNA expression. The two vertical lines represent the differential expression of 2-fold up and down, and the horizontal line represents *p* = 0.05. Thus, the green dots demonstrate the low expression with statistical difference, and the red dots demonstrate high expression with statistical difference.

To explore the molecular mechanism of RF, the RF model of rats was established successfully by the UUO method. The renal tissue samples of the rats from Ctrl and RF groups were collected. The results of Hematoxylin and eosin (HE) and Masson staining demonstrated the injury and fibrosis of renal tissue from RF groups (Figure 1A).

Furthermore, to investigate whether the circRNAs could mediate the RF or not, the experiment of RNA-seq was implemented. A total of 1101 cirRNAs were identified, and 162 DE circRNAs were screened with the criteria |LogFC| > 1 and *p* < 0.05 (Figure 1B) in RF VS. Ctrl, with 59 up-regulated and 103 down-regulated ones respectively (Figure 1C).

**FIGURE 2:**
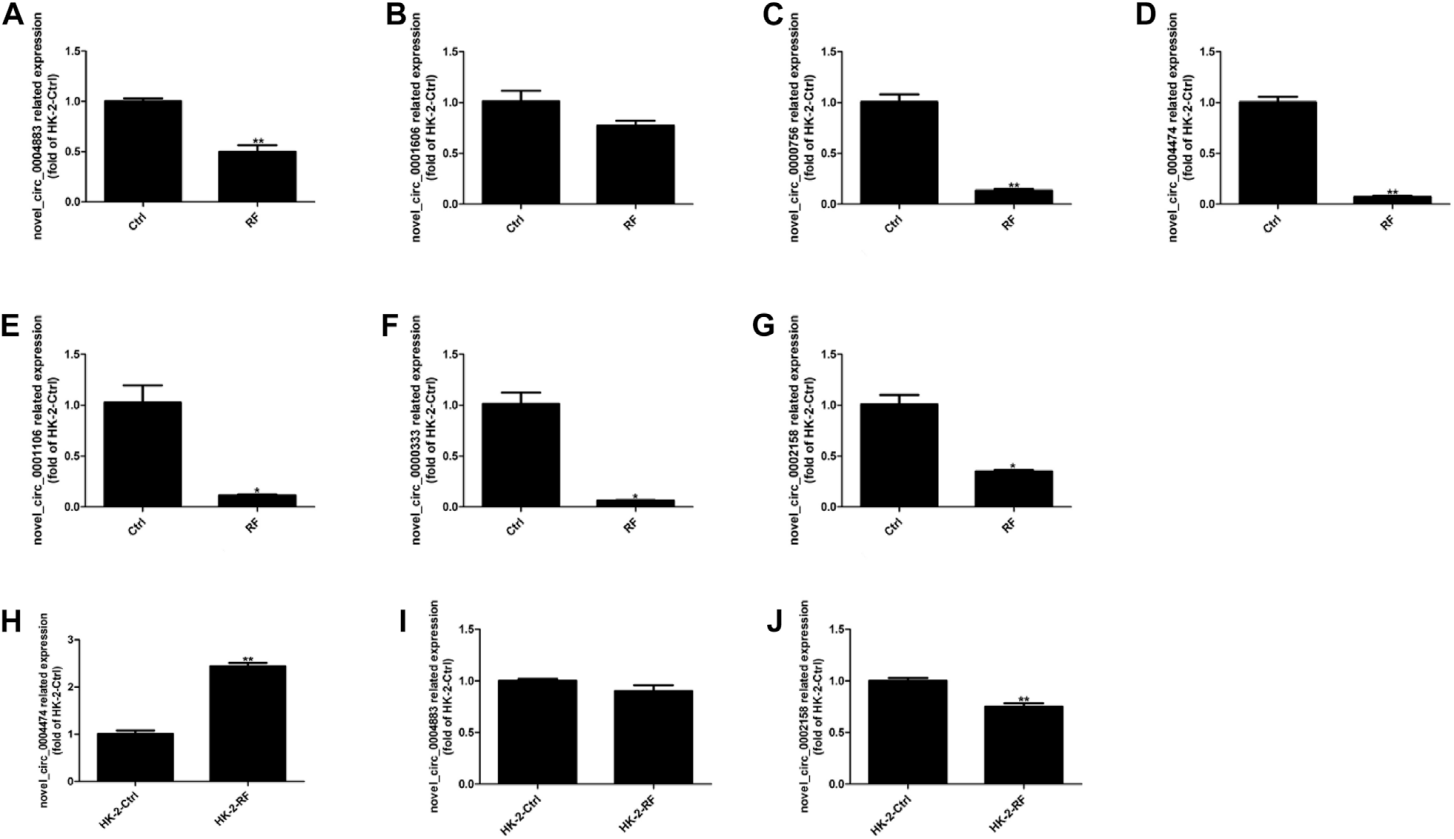
Expression levels of circRNAs were assessed by quantitative reverse transcription polymerase chain reaction (qRT-PCR). **(A–G)** The qRT-PCR results of seven candidate circRNAs indicated the accuracy of RNA-sequencing. **(H–J)** The expression levels of three circRNAs were detected in the cell model of renal fibrosis (RF). Comparisons were carried out with independent sample t-tests, and data have been represented as mean ± SD. *p < 0.05, **p < 0.01, and ***p < 0.001.

Subsequently, to detect the accuracy of the RNA-seq results seven DE circRNAs (circ_0004883, circ_0001606, circ_0000756, circ_0004474, circ_0001106, circ_0000333 and circ_0002158) were selected randomly. The qRT-PCR indicated the results of RNA-seq were accurate and suitable for further analyses (Figures 2A–G).

Interestingly, then we found that circ_0004474, circ_0004883, circ_0002158 were highly homologous to human circRNAs. So, the cell model of RF was established using the human kidney epithelial cell line (HK-2). Thence, homologs of circ_0004474, circ_0004883, circ_0002158 were repeatedly verified by qRT-PCR (Figures 2H–J). Compared to the normal control, the expression level of circ_0002158 of human was significantly reduced in the cells with RF (Figures 2G, J), and similar phenomenon was found in the rat model of RF, suggesting that this novel circRNA might be associated with regulating the occurrence and development of RF. The novel circ_0002158 was first found using this tool. By Blast, we could find homolog (with 89.7% homology) of circ_0002158 in human database. So, we tended to further study for circ_0002158.

### Relationship of circ_0002158 and kidney injury-related factors

**FIGURE 3:**
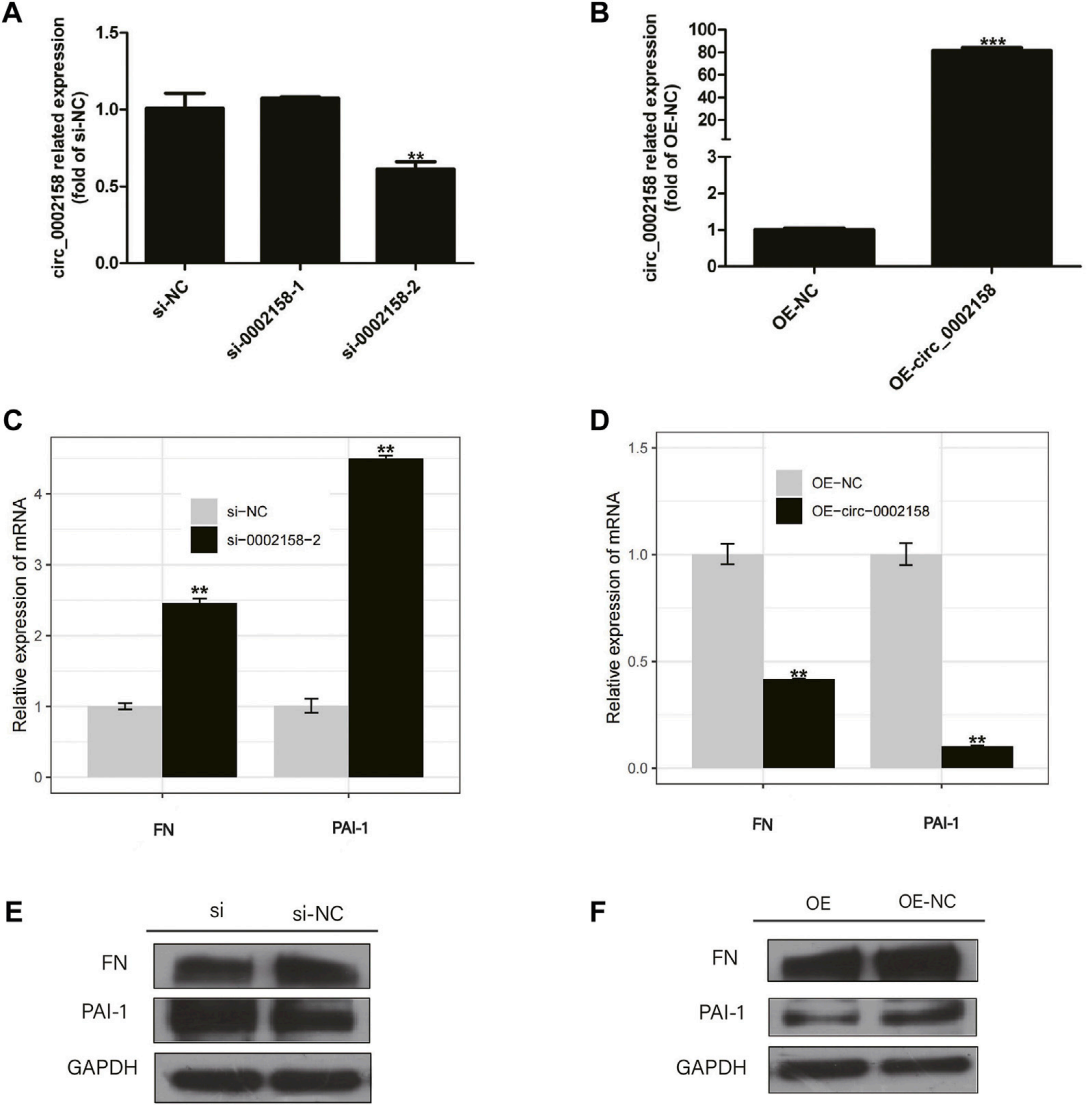
The circ_0002158 regulating expression of fibronectin (FN) and plasminogen activator inhibitor-1 (PAI-1). **(A,B)** HK-2 cells were transfected with siRNAs (circ_0002158–1 and circ_0002158–2), and plasmids containing circ_0002158. The expression levels of circ_0002158 were detected by qRT-PCR. **(C,D)** The expression levels of FN and PAI-1 were detected by qRT-PCR in HK-2 cells transfected with siRNA (circ_0002158–2) or plasmids containing circ_0002158. Comparison was made by independent sample t-tests. *p < 0.05, **p < 0.01, and ***p < 0.001. **(E,F)** The Western blotting analyses were implemented for detecting the expression levels of FN and PAI-1.

HK-2 cells were transfected with plasmids containing circ_0002158, and the siRNAs (circ_0002158–1 and circ_0002158–2). RT-qPCR results showed that the OE and knockdown of circ_0002158 were made successfully in HK-2 cells (Figures 3A,B).

The expression levels of RF related factors, including Fibronectin (FN) and plasminogen activator inhibitor-1 (PAI-1), were detected by qRT-PCR and WB analyses. It showed that the expression levels of FN and PAI-1 were significantly increased in the cells with knockdown of circ_0002158 (Figures 3C, E), but significantly decreased in cells with OE of circ_0002158 (Figures 3D, F), comparing with the normal controls. These results suggested that the circ_0002158 could negatively regulate the expression of FN and PAI-1.

### Circ_0002158 regulates apoptosis and proliferation of cells

**FIGURE 4:**
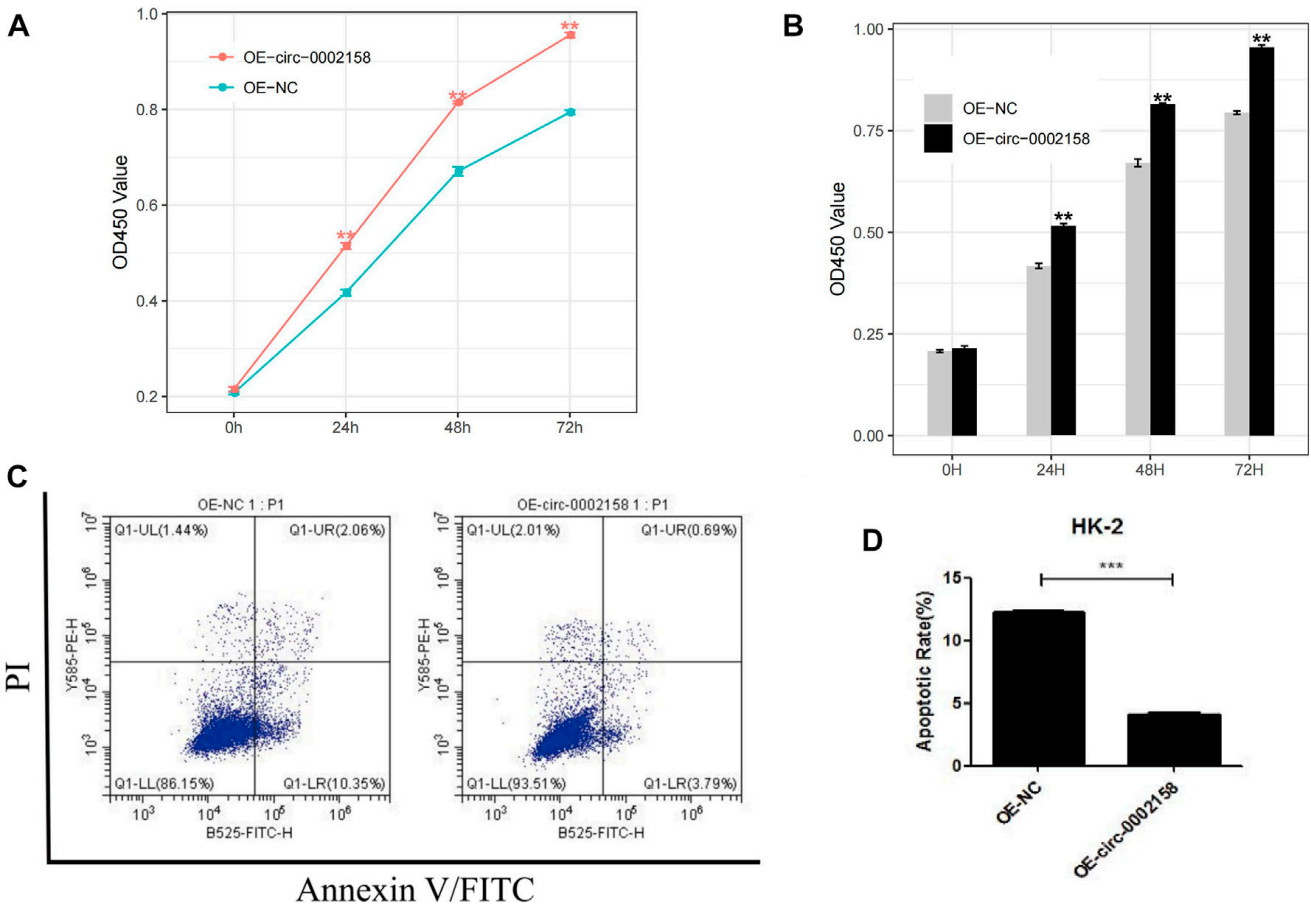
Circ_0002158 regulating the proliferation and apoptosis of HK-2 cells. **(A,B)** Overexpression (OE) of circ_0002158 promoted proliferation of HK-2 cells at 0, 24, 48 and 72 h as assessed using MTT assay. **(C,D)** OE of circ_0002158 function in the HK-2 cells was assessed using flow cytometry. Data have been represented as mean ± SD. *p < 0.05, **p < 0.01, and ***p < 0.001.

To further explore whether the circ_0002158 regulates the proliferation of HK-2 cells or not, the CCK-8 arries were conducted for HK-2 cells from OE and NC groups. The results indicated that viability cells with OE of circ_0002158 was increased significantly, at different time points (0, 24, 48 and 72 h) (*p* < 0.001) **(** Figures 4A, B).

Furthermore, flow cytometry was used to determine whether circ_0002158 could affect apoptosis of HK-2 cells or not (Figure 4C). It displayed that the OE of circ_0002158 significantly inhibited apoptosis of cells, and the number of apoptotic cells obviously reduced about 66.7%, comparing with the NC group (Figure 4D). In all, above results indicated that circ_0002158 had the capacities to regulate proliferation and apoptosis of the HK-2 cells.

### Regulative mechanism of circ_0002158

To explore the molecular mechanism of circ_0002158 mediating the proliferation and apoptosis of HK-2 cells, iTRAQ analysis was applied to uncover altered protein expressions and signaling pathways, using the cell samples from OE and NC groups.

**FIGURE 5:**
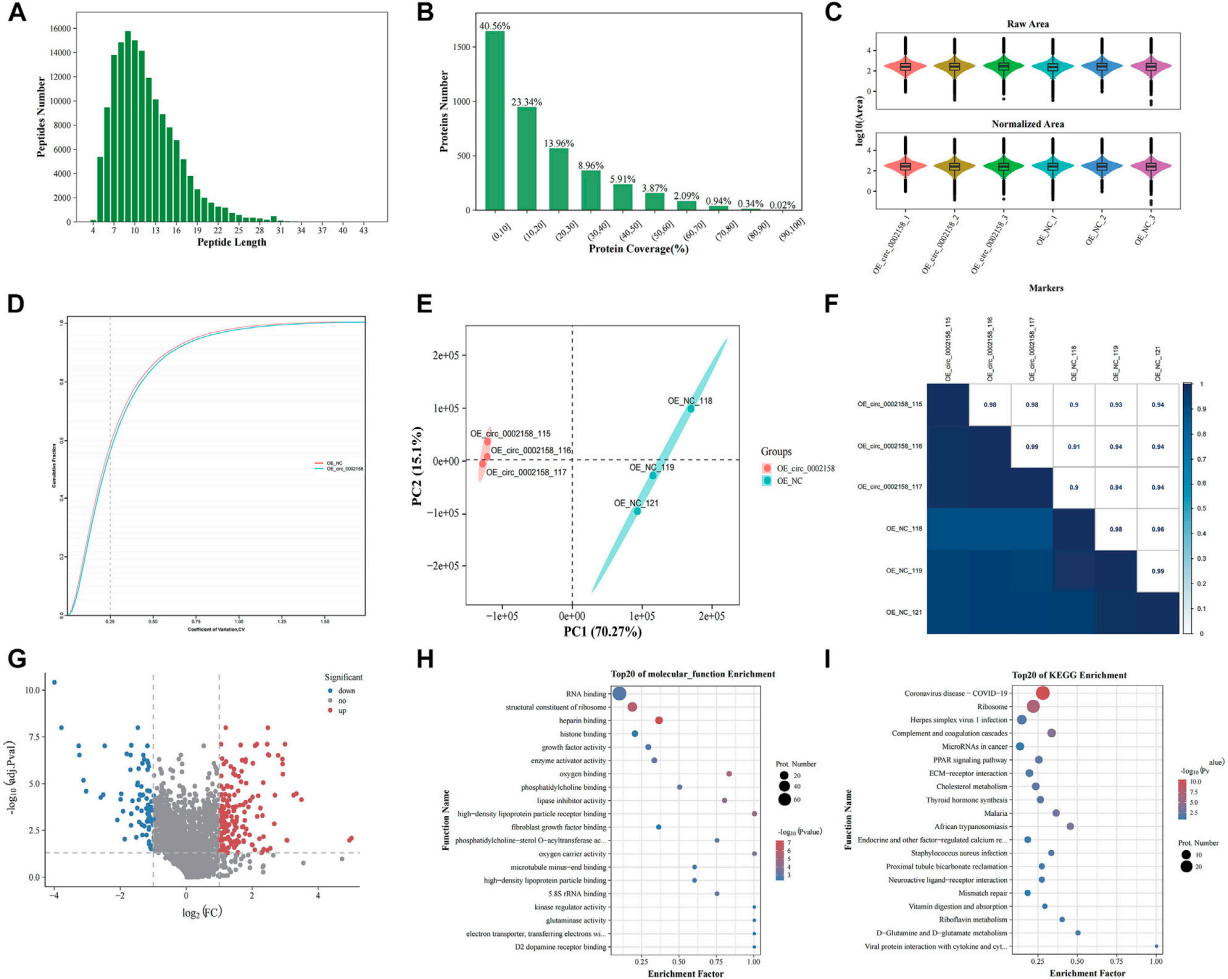
iTRAQ proteomics analysis. **(A)** Length distributions of peptides. **(B)** Protein identification coverage distribution map. **(C)** Abundance map of the sample. **(D)** Sample repeatability plot. **(E)** Principal component analysis plot. **(F)** Pearson correlation coefficient plot for samples. **(G)** Volcano plots showing the significant up-regulated and down-regulated differentially expressed proteins (DEPs). Gene ontology **(H)** and Kyoto Encyclopaedia of Genes (KEGG) terms **(I)** of host genes from which DEPs were identified.

In total, the quality of the data obtained from the iTRAQ was analyzed using parameters such as coefficient of variation about repeatability, distribution of unique peptide, peptide length, and distribution of coverage (Supplementary Table S1). From, the length of the identified peptides is mainly between 5 and 17, which is consistent with the molecular weight range (400–1200 Da) identified by the mass spectrometer (Figure 5A). The distribution map of protein identification coverage results showed that the coverage of a large number of proteins were below 20%, indicating that the overall accuracy of the identification results was highly (Figure 5B). The abundance map showed that there is no significant difference in the total protein expression of the 12 samples (Figure 5C). The repeatability map showed that the experiment is reproducible (Figure 5D). A principal component analysis (PCA) diagram and Pearson correlation coefficient (PCC) plot for the sample can be seen that the two groups of samples can be distinguished (Figures 5E,F).

A total of 224 DEPs were found in OE VS*.* NC according to FC and *p*-value (FC ≥ 1.2 or ≤0.83, p ≤ 0.05) (Figure 5G), and the up-regulated and down-regulated ones were 142 and 82 (Supplementary Table S2), respectively. The FN was one of down-regulated proteins.

Moreover, GO functional enrichment of these DEPs was carried out. The mainly enriched terms, include “heparin binding” (GO: 0008201), “structural constituent of ribosome” (GO: 0003735), “growth factor activity” (GO: 0008083), “kinase regulator activity” (GO: 0019207) and “high-density lipoprotein particle binding” (GO: 0008035) (Figure 5H), which were related to RF or extracellular matrix (ECM) deposition.

In addition, KEGG enrichment for DEPs was implemented. The multiple enriched pathways, including “Coronavirus disease - COVID-19” (ko05171), “Ribosome” (ko03010), “Complement and coagulation cascades” (ko04610), “MicroRNAs in cancer” (ko05206) and “ECM-receptor interaction” (ko04512) (Figure 5I), which were related to diseases and ECM deposition.

On basis of GO and KEGG enrichment results, we found that some node genes were DE between the OE and NC groups. For example, thrombospondin 1 (THBS1), vitronectin (VN) and FN genes were down-regulated in the OE group. Among them, FN is also consistent with our previous experiments, and these three genes are all related to ECM. In addition, down-regulation of the expression of transforming growth factor beta 2 (TGF-β2) associated with CKDs, was detected in the OE group.

## Discussion

A variety of non-coding RNAs, including microRNAs (miRNAs), long non-coding RNAs (lncRNAs) and circRNAs, have been indicated to medeiate RF by regulating expression levels of mRNAs. Recent investigations have implicated aberrantly expressed lncRNAs were associated with development and progression of RF, suggesting that lncRNAs as diagnostic and/or prognostic biomarkers might play a crucial role in determining the clinical manifestation of RF (Xia et al., 2021). For example, the OE of lncRNA HOTAIR down-regulates expression of miR-124 to activate the Notch1 pathway, hence promoting EMT in TGF-β1-induced HK-2 cells and RIF in UUO rats (Zhou et al., 2019). The OE of lncRNA 1700020I14Rik reduces the cell proliferation and expression of several genes (Col-4, FN, TGF-β1) associated with fibrosis by directly interacting with miR-34a-5p in a high glucose (HG) environment (Li et al., 2018). Although circRNAs have been recognized as vital regulators in multiple diseases, few studies have revealed that the abnormal expressions of circRNAs are related to the RF (Fan et al., 2021; Yi et al., 2021; Cheng et al., 2022). Hence, the role and regulatory mechanism of circRNAs in RF need urgently to be uncovered. Thence in the current study, we for the first time found that circ_0002158 was associated with the RF. And also, previous studies showed abnormally expressed circRNAs are related with the cells proliferation and apoptosis (Liu et al., 2018; Yuan et al., 2019; Shi et al., 2020; Mo et al., 2021). CircRNAs can act as sponges for miRNAs to modulate gene expression, thereby exerting a competitive endogenous RNAs (ceRNAs) (Gu et al., 2017; Chen et al., 2019; Fang et al., 2019; Kristensen et al., 2019). We demonstrated that OE of circ_0002158 could promote proliferation and inhibit apoptosis by mediating expression of FN and PAI-1 cells to ameliorate the progression of RF in HK-2 cell, while the knockdown of circ_0002158 led to opposite effects.

In general, the ECM consists mainly of FN, collagens (including types I and IV), laminin, and proteoglycans (Kolset et al., 2012). The ECM accumulates after injury and promote the fibrosis by increasing systemic PAI-1 in fibroblasts. To date, increasing evidence indicates that FN and PAI-1 acts as a marker for the development of RF (Yao et al., 2019; Zheng et al., 2019). Consistently, we found that the expression level of circ_0002158 was significantly reduced HK-2 cells with RF, and FN and PAI-1 was significantly reduced in HK-2 cells with OE of circ_0002158. It could be speculated that circ_0002158 might constitute potential ceRNA axes by sponging various miRNAs to regulate the expression of some genes (FN and PAI-1) associated with RF. In the future, the discoveries of ceRNA axes could contribute to ameliorating the progression of RF.

Moreover, GO and KEGG enrichment data revealed that down-regulation of ECM-related genes, which were verified by foregoing experiments. Since ECM deposition is the main factor inducing RF formation, we wondered whether circ_0002158 might improve the progression of RF by reducing the level of ECM deposition. Furthermore, relevant growth transforming factors may also be involved in this process. The molecular mechanism of circ_0002158 to improve RF is also the direction of our subsequent research.

Collectively, our present study found that circ_0002158 played an anti-fibrosis role in human RF. Moreover, we first found that circ_0002158 may regulate multiple expressions of gene involved in RF. Thus, the data suggest that circ_0002158 might contribute to treatment for RF disease.

## Data Availability

The datasets presented in this study have been submitted to online repositories. The transcriptomic data can be obtained from NCBI with accession No: PRJNA895745, while proteomic data can be obtained from ProteomeXchange with accession No: PXD037827.
